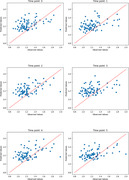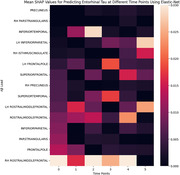# Predicting Future Entorhinal Tau Levels through Amyloid PET Patterns: A Longitudinal Machine Learning Approach in Preclinical Alzheimer’s disease

**DOI:** 10.1002/alz.093364

**Published:** 2025-01-09

**Authors:** Amirali Vahid, Jafar Zamani, Hadi Hosseini

**Affiliations:** ^1^ Stanford University, Stanford, CA USA

## Abstract

**Background:**

Amyloid‐β (Aβ) plaques and tau tangles represent pivotal hallmarks of preclinical Alzheimer's disease (AD). Accumulating studies highlight a potential association between Aβ and tau, suggesting that the progressive deposition of Aβ may significantly contribute to build up of tau in the entorhinal cortex which subsequently result in neurodegeneration and cognitive decline. However, pattern of Aβ depositions that would result in tau development or propagation remains elusive. This study aims to leverage advanced machine learning along with longitudinal PET measures of brain Aβ and tau load to identify the most critical brain regions where the presence of Aβ is predictive of levels of future entorhinal tau levels.

**Method:**

An Elastic‐Net model was applied to baseline Aβ SUVR data to predict entorhinal tau levels across multiple tau‐PET acquisition time points (0 to 5 years from baseline). The study focused on 80 subjects (41 female, mean (SD) age 71.8 (6.7) years) from the Alzheimer’s Disease Neuroimaging Initiative (ADNI) who had longitudinal data at all specified time points. Additionally, explainable AI, particularly SHapley Additive exPlanations (SHAP), was utilized to discern the model's learning process and identify brain regions significantly contributing to predicting the longitudinal entorhinal tau levels.

**Result:**

The R‐scores between actual and predicted entorhinal tau value for time points 0 to 5 were: 0.36, 0.42, 0.47, 0.38, 0.47, and 0.40 (all p‐values < 0.001), as depicted in Figure 1. Notably, SHAP measures revealed that Aβ load in the rostral middle frontal, superior frontal and frontal pole regions contributed the most to prediction of future entorhinal tau 0 to 4 years later (Figure 2). These regions accompanied with the left inferior parietal lobe as most significant predictors of entorhinal tau at time point 5 (Figure 2).

**Conclusion:**

Our findings suggest that Aβ loud in the medial and rostral frontal regions are highly predictive the development of tau in the entorhinal cortex within four years and may signal progression to clinical AD, considering that entorhinal tau is the most proximal correlate of neurodegeneration and future cognitive decline